# PAD-S/CSA as a candidate shared representation layer for computational psychotherapy: minimal architecture and a staged validation roadmap

**DOI:** 10.3389/fpsyt.2026.1817747

**Published:** 2026-04-29

**Authors:** Eik Niederlohmann

**Affiliations:** Department of Psychosomatic Medicine and Psychotherapy, Kliniken Erlabrunn, Breitenbrunn, Germany

**Keywords:** computational psychiatry, computational psychotherapy, CSA, PAD-S, psychotherapy process coding, shared representation layer, state-space model (SSM), transdiagnostic functioning

## Abstract

Psychotherapy schools often describe overlapping process phenomena in non-interoperable vocabularies. This pluralism is clinically valuable but computationally costly: datasets become difficult to compare, clinically load-bearing distinctions are collapsed into convenience labels, and artificial intelligence (AI) systems inherit annotation schemes rather than a clinically interpretable intermediate representation. Building on the Perceive–Assess–Dose–Safeguard (PAD-S) framework and the Conflict-Square Algorithm (CSA), this theory article asks a narrower question than the prior PAD-S and CSA papers: can the same variables be formulated as a candidate shared representation layer between heterogeneous observation models and school-specific intervention policies? The proposed layer projects a high-dimensional biopsychosocial state into four clinically observable process coordinates—defensive/avoidant organization (DEF), anxiety/arousal and tolerance (ANX), progression toward direct experience and action (PRO), and self-attack/shame processes (SUP)—plus a safety threshold that constrains admissible intervention intensity. The contribution is architectural rather than empirical: it isolates the representational role from earlier decision-grammar and transcript-coding roles; clarifies the distinction between observations, representation, and policy; specifies a minimal falsifiable family of state-transition models; illustrates translation across four pragmatic therapy families; and defines a staged validation order from reliability and function linkage to transcript-level predictive operationalization and only then sparse equation discovery. The framework should therefore be read as a candidate shared representation layer for computational psychotherapy and computational psychiatry rather than as a therapy protocol, a fitted predictive model, a complete generative theory, or an autonomous decision system. No new dataset, fitted classifier, transcript-level predictive result, or discovered equation is reported here. The article aims instead to state what would count for or against PAD-S/CSA as a clinically interpretable interface for later empirical modeling.

## 1 Introduction

Clinical mental health care must continuously integrate at least three levels of description: symptoms and diagnoses, psychological functioning and role participation, and moment-to-moment safety in emotionally charged interpersonal exchanges. Categorical diagnosis remains indispensable, but it is often insufficient for deciding how much interpersonal or affective load can be introduced safely, how to understand rupture and repair in real time, or how to formulate function-oriented goals in severe, chronic, or mixed presentations. In parallel, computational psychiatry has set itself the task of linking neurobiology, environment, and symptoms in formal terms, ideally without collapsing the person into biology alone (1–3). This creates a practical problem: clinically rich process information is abundant, yet much of it is stored in school-specific vocabularies that do not translate well into machine-readable representations.

The result is a bottleneck in computational psychotherapy. If a research group codes one dataset in the language of defenses, another in the language of schemas, another in emotion regulation terms, and another in generic sentiment or dialogue-act labels, model comparison becomes conceptually muddy before it becomes statistically difficult. In this setting, high-performing AI can still be clinically shallow because it predicts labels that were convenient to annotate rather than variables that clinicians would trust to guide high-stakes decisions. Human-centered AI requires the opposite orientation: the representation should be clinically interpretable first and then computationally tractable second (4, 5).

Perceive–Assess–Dose–Safeguard (PAD-S) and Conflict-Square Algorithm (CSA) were developed in response to this problem. PAD-S introduced a safety-gated state-action grammar for psychotherapy micro-decisions and already contained germinal representation-layer language. CSA extended that logic into transcript-oriented episode coding, pseudocode, annotated examples, Mini-ICF-APP crosswalks, and a feasibility-oriented documentation framework (8, 9). The present article, therefore, does not claim to invent PAD-S variables, transcript coding, or SPICE (Sparse, Interpretable Cognitive Equations) relevance *de novo*. Its narrower claim is that PAD-S/CSA can be analyzed and specified primarily as a candidate shared representation layer between heterogeneous observation models and school-specific intervention policies, allowing different schools to remain theoretically plural while becoming more commensurable, auditable, and model-ready.

The need for such a representation layer is amplified by the current AI landscape. Natural-language and multimodal models can classify emotions, symptoms, and conversational patterns with increasing accuracy, including in psychotherapy-related material (32, 33). Nevertheless, model accuracy does not by itself solve the representational problem. If the target labels do not preserve clinically salient distinctions such as safety gating, defensive avoidance, or self-attack following progress, the resulting system may remain useful for transcription or summarization while still failing as a meaningful clinical interface. The representational question is therefore prior to many model-selection questions.

This article makes four narrower contributions. First, it isolates the representational role of PAD-S/CSA from its earlier decision-grammar and transcript-coding roles. Second, it offers a minimal formalization in which a high-dimensional biopsychosocial state is projected into four clinically observable process coordinates plus a discrete safety threshold. Third, it clarifies the relationship between representation and school-specific policy so that psychotherapy pluralism can be preserved rather than flattened. Fourth, it defines a staged validation order moving from reliability and function linkage to transcript-level predictive operationalization and only then to sparse equation discovery. The goal is not to claim that the four coordinates are already the one true ontology of psychological functioning but to propose a minimal, falsifiable, clinically interpretable interface that can be tested, revised, or rejected.

## 2 Scope, stance, and differentiation from prior PAD-S/CSA work

This article adopts a deliberately narrow stance. It does not offer psychometric closure for the four coordinates, a unified theory of psychotherapy, a complete computational psychiatry model, a decision-support evaluation, or a manualized sequence of therapeutic moves. What it contributes instead is the representational and dynamical architecture: a candidate shared representation layer plus an admissible family of transition models. Empirical implementation choices—segmentation granularity, coding density, baseline predictive model class, regularization, and held-out evaluation design—are intentionally deferred to later empirical work. In this respect, the paper is best read as a hypothesis and theory contribution with an explicit validation agenda, not as a finished, fitted model.

In the prior PAD-S/CSA literature, the framework has appeared in at least three roles: as a descriptive clinical language, as a safety-gated micro-decision grammar, and as a transcript-oriented coding/documentation scheme linked to function (8, 9). Those roles are compatible, but they are not identical. The present article gives primacy to a fourth task: evaluating PAD-S/CSA as a candidate shared representation layer between observation models and school-specific policies. A school may map its own constructs into PAD-S/CSA and continue to use its own policy for selecting interventions. PAD-S decision matrices remain possible, but they are optional rather than constitutive here.

Originality here should therefore be read as role differentiation rather than vocabulary novelty. PAD-S already contained orientation-translatable, machine-readable language in germinal form, and CSA already operationalized transcript coding, function linkage, and feasibility-oriented documentation. What is newly foregrounded here is the representational question itself: whether the same variables can serve as a low-dimensional interface between heterogeneous observations and school-specific actions, under clearly stated non-goals, a cleaner variable hierarchy, a minimal falsifiable model family, a dyadic extension, and a staged validation order. **Table 1** summarizes what is newly isolated or formalized in the present manuscript relative to the earlier papers. The contribution is architectural and methodological, not empirical.

**Table 1:** Newly foregrounded or formalized contributions in the present manuscript relative to the prior PAD-S and CSA papers.

Publication	Primary focus	Level of analysis	What is newly foregrounded or formalized here
Niederlohmann 2026 PAD-S (8)	Safety-gated psychotherapy micro-decision grammar; explicit computational psychiatry bridge; transcript relevance noted.	Moment-to-moment state-action logic.	Isolates the representational role from the micro-decision grammar; defines the observation–representation–policy hierarchy, explicit non-goals, minimal transition family, and staged validation order.
Niederlohmann 2026 CSA (9)	Four-node transcript-oriented framework with pseudocode, annotated transcripts, and Mini-ICF-APP linkage.	Transcript coding, functional diagnosis, and documentation.	Preserves transcript coding and function linkage from CSA but adds the architectural question itself: whether PAD-S/CSA can function as a shared layer across observation models and school-specific policies, plus dyadic extension and explicit falsification criteria.
Present manuscript	Candidate shared representation layer with explicit non-goals and evidential boundaries.	Representation layer between observation models and intervention policies.	Architectural contribution: role differentiation, cleaner variable hierarchy, restricted model family, dyadic extension, and staged validation order.

PAD-S, Perceive–Assess–Dose–Safeguard; CSA, Conflict-Square Algorithm.

## 3 Why these four coordinates?

A proposal of this kind risks arbitrariness if defensive/avoidant organization (DEF), anxiety/arousal and tolerance (ANX), progression toward direct experience and action (PRO), and self-attack/shame processes (SUP) are presented as an already-validated latent factor structure. That is not the claim. The claim is narrower: these four coordinates satisfy a set of design criteria that make them candidate coordinates for a shared process representation in psychotherapy and computational use. The criteria are clinical observability at the episode level, direct relevance for dosing and safety, transdiagnostic applicability, translatability across orientations, and plausible linkage to functioning in the ICF/Mini-ICF-APP sense (16–18). Put differently, the proposal is engineering-like before it is taxonomic: the coordinates are selected because they preserve clinically consequential distinctions while remaining sparse enough to model.

The first two coordinates, DEF and ANX, emerge from a long psychodynamic and emotion-process tradition in which defensive avoidance and affect tolerance jointly determine whether direct experiential work is possible (10–13). Across affect-phobia, short-term dynamic, and relational traditions, the core process logic is recurrent: conflictually meaningful material becomes imminent, arousal rises, defenses organize access to that material, and the clinician must decide whether to deepen, regulate, or redirect. That basic logic can be observed without requiring allegiance to any one school’s vocabulary. A cognitive behavioral therapy (CBT) or schema-trained clinician may talk about avoidance, deactivation, overcontrol, rumination, experiential avoidance, or coping modes rather than defenses, but the process function remains mappable.

PRO is necessary because low anxiety and low defense do not by themselves tell us whether constructive movement is occurring. A session can become quieter without becoming more alive, more agentic, or more action-oriented. PRO captures forward movement toward direct experience, need articulation, value-consistent action, fuller contact, or more adaptive participation. Conversely, SUP is necessary because deterioration is often not reducible to anxiety alone. Patients may move forward and then immediately attack themselves, collapse pride into shame, moralize needs, or convert relief into self-punishment. In fragile and severe presentations, this can be the decisive process variable. Frederickson’s training work emphasizes exactly this need to track how progress is followed by punishment or withdrawal and to protect positive states rather than assuming that more intensity is always therapeutic (12, 13).

These coordinates are therefore not presented as psychodynamic essentials or fixed natural kinds. They are candidate process invariants. DEF indexes organized avoidance; ANX indexes arousal and tolerability; PRO indexes approach, agency, and workable movement; SUP indexes punitive self-relation and shame attack. The set is intentionally minimal and probably incomplete. It does not claim to exhaust attachment, identity, mentalization, culture, or symptom phenomenology. Instead, it claims to be a compact set of clinically action-relevant process coordinates that can be tested prospectively. Classical factor analysis on cross-sectional questionnaire items would be a weak test of this proposal because the coordinates are defined as time-varying process variables rather than as trait scales. Better tests are the reliability of episode coding, out-of-sample predictability of state transitions, function linkage, and the ability of sparse models to recover a stable interaction structure. The burden of proof is therefore comparative rather than metaphysical: if another low-dimensional set proves more reliable, more function-linked, or more stable under sparse recovery, it should replace or revise the present one.

## 4 Minimal formalization

### 4.1 High-dimensional state, observations, and context

Let x_t_ denote the person’s latent high-dimensional biopsychosocial state at time t. The dimensionality of x_t_ is intentionally left large because the point of the present article is not to deny complexity but to compress it. x_t_ may include symptom variables, bodily states, action tendencies, relational expectations, contextual affordances, memories, habits, role demands, and other clinically relevant determinants. The representation layer is not assumed to recover the full state; it is assumed to preserve a clinically useful projection of it.

The use of the term biopsychosocial is deliberate and follows Engel’s classic argument that clinically adequate models must integrate biological, psychological, and social levels rather than reducing one to the others (15).

To keep the notation unambiguous, a single observation symbol, o_t_, is used. o_t_ denotes the currently available multimodal observations—speech, prosody, posture, turn-taking, reportable bodily cues, therapist observations, and other coded interactional signals. Context is represented separately as c_t_ and includes setting, institutional demands, family or dyadic configuration, phase of treatment, and other external constraints. A generic observation equation is as follows:

o_t_ = h(x_t_, c_t_) + ν_t_.

The function h is not assumed to be linear or complete. It simply expresses that what can be observed is a context-shaped transform of a much richer underlying state. Clinically, the therapist never works directly on x, but on what is inferable from o_t_ in a setting c_t_. The operational claim is therefore not that P is uniquely identifiable from observation but that clinically useful inferences about z_t_ can be made with explicit uncertainty and auditable coding rules.

### 4.2 Projection into PAD-S state and safety threshold

The representational act is a projection P from the high-dimensional latent state into a low-dimensional coordinate vector z_t_:

z_t_ = P(x_t_) = [DEF_t_, ANX_t_, PRO_t_, SUP_t_]^T^.

Each element of z_t_ is interpreted as a process coordinate rather than a trait score. Depending on the operationalization, the coordinates may be coded categorically, ordinally, or continuously. In transcript-based work, the practical unit may be a turn or an episode line; in multimodal work, it may be a short time window. A discrete safety threshold τ_t_ then captures whether the current configuration is workable, cautioned, or non-workable for intensifying intervention:

τ_t_ = g(z_t_, c_t_), τ_t_ ∈ {A, B, C}.

Here, A denotes a workable range, B a narrowed or cautionary range, and C a stop/de-escalation range. The threshold is framed as a safety constraint, not a pathology label. It regulates admissible action sets:

a_t_ ∈ A(τ_t_).

Thus, the therapist’s next move a_t_ is constrained by the current safety zone. PAD-S/CSA is therefore not only descriptive; it also marks whether certain action classes are presently admissible under a safety constraint. This does not make the threshold a treatment algorithm on its own because the choice among admissible actions remains policy-dependent. **Table 2** summarizes the notation used in the minimal formalization.

**Table 2:** Glossary for the minimal formalization used in this manuscript.

Symbol	Meaning	Status in this article	Illustrative example
x_t_	High-dimensional latent biopsychosocial state	Theoretical background state; not directly observed	Symptoms, bodily arousal, role pressures, expectations, and habits
o_t_	Current multimodal observations	Observed/coded data	Speech, pauses, gaze, prosody, bodily markers, and coded transcript features
c_t_	Context	Observed or externally specified	Setting, family context, treatment phase, and institutional demand
z_t_	PAD-S state vector [DEF, ANX, PRO, SUP]^T^	Shared representation layer	Current balance of avoidance, arousal, progression, and self-attack
τ_t_	Safety threshold/zone	Discrete safety constraint	A workable, B caution, and C stop/de-escalate
a_t_	Clinician/system action	School-specific policy output	Clarify defense, regulate anxiety, deepen, protect positives, and meta-comment
s_t_	Dyadic safety/felt security	Additional relational latent variable	Alliance strength, felt safety, and co-regulatory stability
P(·)	Projection operator	Representation mapping	Compression of high-dimensional functioning into four coordinates
π^T^(·)	School-specific policy	Optional action rule	How one therapeutic orientation maps states to interventions

PAD-S, Perceive–Assess–Dose–Safeguard; DEF, defensive/avoidant organization; ANX, anxiety/arousal and tolerance; PRO, progression toward direct experience and action; SUP, self-attack/shame processes.

### 4.3 A minimal falsifiable family of state-transition equations

Any candidate computational interface becomes unconvincing if it names variables without specifying what would count as an admissible model family. I therefore introduce a deliberately minimal discrete-time family. It is not intended as the only eventual specification; it is intended as the smallest family that makes state, action, and context effects explicit while remaining estimable in principle:

z_t_₊_1_ = A z_t_ + B a_t_ + C c_t_ + D(z_t_ ⊗ a_t_) + E(z_t_ ⊗ c_t_) + ξ_t_.

A, B, and C capture the main effects of the current state, action, and context, respectively. D and E capture state-by-action and state-by-context interactions via the tensor/Kronecker product ⊗, respectively. The equation is deliberately minimal: it omits within-state and higher-order terms from the headline form for readability, not because such terms are impossible. Those richer interactions belong in later candidate libraries and should be admitted only when they improve held-out performance and remain clinically interpretable.

For sparse continuous-time recovery, the corresponding form can be written as follows:

ż_t_ ≈ Θ(z_t_, a_t_, c_t_)β + ξ_t_.

Here, Θ(·) is an explicitly documented candidate library, and β is a sparse coefficient vector to be identified. In a later empirical implementation, this library could include intercepts; main effects for DEF, ANX, PRO, and SUP; action and context indicators; selected within-state interactions (e.g., DEF × ANX or PRO × SUP); state-by-action terms; threshold-gated terms derived from τ_t_ = g(z_t_, c_t_); and, where coded, optional dyadic-safety terms involving s_t_. The present article does not estimate β; it specifies the class of later models that would make the architectural claim genuinely falsifiable.

Operational discipline matters as much as notation. For transcript-based work, the natural held-out unit is the transcript or case rather than shuffled turns, and model selection should rely on conservative transcript-level comparison. The framework is falsifiable in at least four ways. First, episode-level coding may fail to achieve adequate interrater agreement. Second, sparse transition models based on z_t_, a_t_, and c_t_ may fail to generalize out of sample or may yield mutually incompatible sparse solutions with similar accuracy. Third, the coordinates may show no meaningful linkage to ICF/Mini-ICF-APP functioning. Fourth, translation across therapy families may prove too lossy to be useful. Any of these outcomes would weaken the claim that PAD-S/CSA is a viable shared representation layer.

### 4.4 Representation versus school-specific policy

A central clarification of the present paper is the distinction between representation and policy. The representation layer is z_t_ (and τ_t_): a compressed description of the current process state and its safety constraints. A therapy school’s policy is π_s_, mapping observations or states into actions. This paper, therefore, specifies the representational layer and an admissible model family, while leaving empirical implementation choices—segmentation unit, candidate library, regularization strategy, baseline predictive model class, and transcript- or case-held-out evaluation—to later work. Different schools may share the same representational substrate while still disagreeing about action policy. This is exactly what a pluralistic computational interface should allow.

Formally, one may write a school-specific policy as a_t_ = π_s_(o_t_, z_t_, τ_t_, c_t_), where s indexes the therapeutic orientation. PAD-S decision matrices are one possible policy family, but they are not logically identical with the representation layer itself. In the present formulation, PAD-S/CSA is primarily the interface; action rules are secondary, orientation-dependent, and open to empirical comparison.

### 4.5 Relation to richer generative models

PAD-S/CSA, as specified here, is not itself an active-inference model or any other full generative treatment model. It does not define priors, likelihoods, precision updates, prior preferences, or a free-energy objective. No claim of active-inference implementation is therefore made.

What can be claimed more modestly is that z_t_ and τ_t_ could, in principle, serve as low-dimensional state and constraint variables inside richer generative models developed elsewhere, including active-inference variants (21–23). That possibility is a compatibility claim about interface variables, not a result of the present paper.

## 5 Cross-orientation translation and multi-agent embedding

### 5.1 Four pragmatic therapy families and the meaning of contextual–relational

Psychotherapy pluralism becomes computationally tractable only if overlap and difference are both acknowledged. The literature on common factors and therapeutic alliance suggests real cross-school convergence (6, 7), while school-specific theories and techniques remain non-trivial (10–14, 18–20). The aim of a shared representation layer is therefore not to erase differences but to create a common coordinate system in which differences can be described more precisely.

For practical purposes, I use four pragmatic therapy families. The psychodynamic family includes short-term dynamic, EDT, affect-phobia, and relational psychodynamic traditions (10, 11). The CBT family includes conventional CBT-, DBT-, and schema-informed variants that track coping, modes, skills, and learning contingencies. The systemic family includes family- and couple-oriented work that emphasizes interaction loops, role structure, and circular causality. Finally, I use the label contextual–relational for therapies that emphasize meaning, values, openness to experience, and the immediate relational field. This umbrella may include contextual-behavioral work, such as Acceptance and Commitment Therapy (ACT) or Functional Analytic Psychotherapy (FAP), but also experiential-relational approaches such as Emotion-Focused Therapy (EFT), Accelerated Experiential Dynamic Psychotherapy (AEDP), and person-centered experiential work when they function clinically as context-sensitive relational approaches (12–14). The term is therefore pragmatic, not canonical: it is offered to clarify scope, not to impose a new taxonomy.

**Figure 1:**
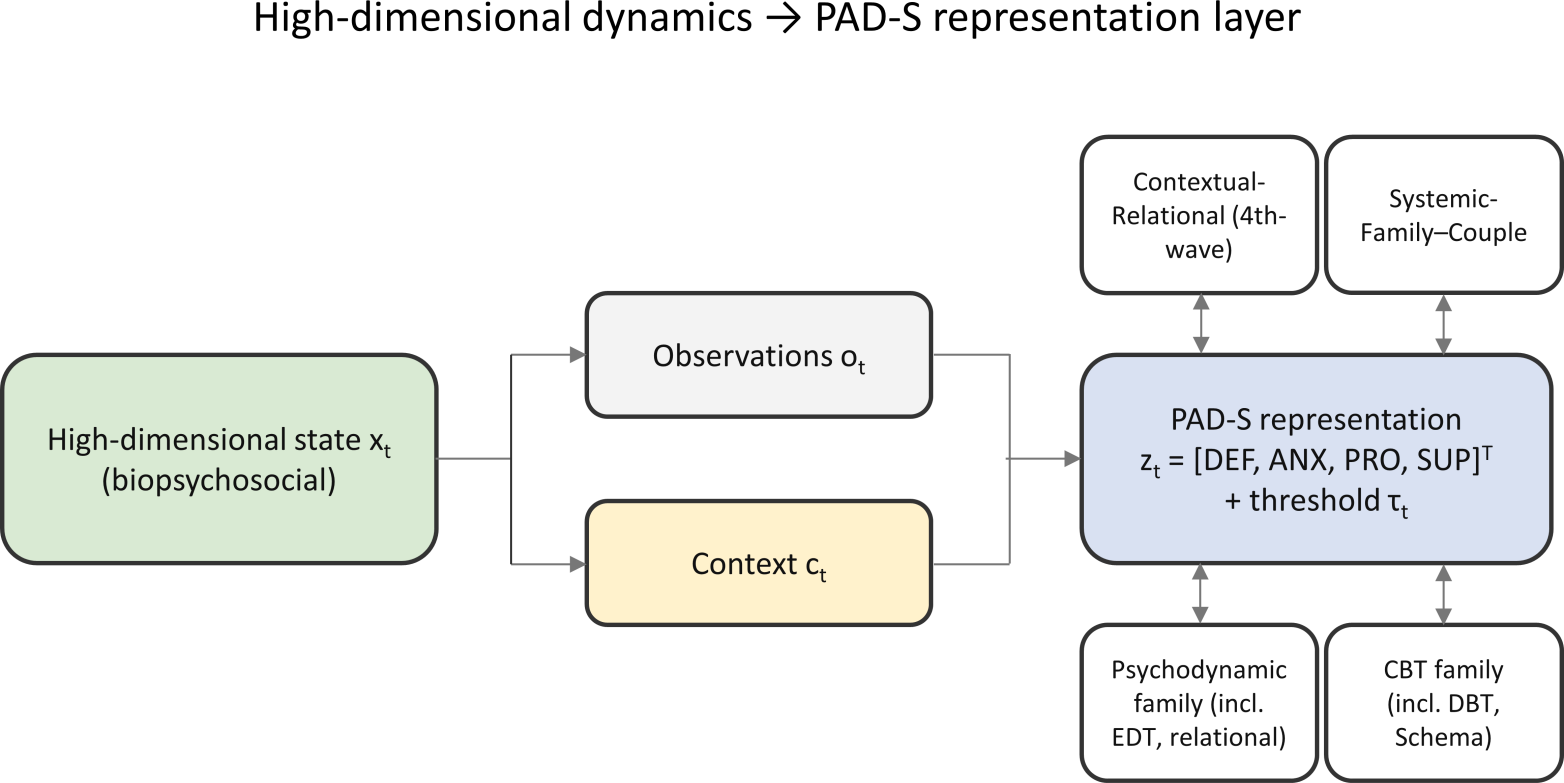
High-dimensional biopsychosocial state projected into a PAD-S representation layer. The four surrounding boxes indicate four pragmatic therapy families, each of which can be understood as a partial low-dimensional “touch” of the same person-in-context system. The label contextual–relational is used pragmatically to cover context-sensitive, values-oriented, and experiential-relational approaches rather than as a fixed official taxonomy. PAD-S, Perceive–Assess–Dose–Safeguard.

**Figure 1** illustrates the core idea. Each family can be understood as a partial, low-dimensional “touch” of the same person-in-context system. **Table 3** then gives an operational translation sketch showing how the four coordinates can be rendered in the idiom of each family. The table is intentionally illustrative and lossy: it shows mappability, not full equivalence of formulations.

**Table 3:** Illustrative translation of the four PAD-S coordinates across four pragmatic therapy families.

Family	DEF	ANX	PRO	SUP
Psychodynamic/EDT	Defense, avoidance, resistance, detour, and transference resistance	Affect tolerance, striated vs smooth muscle arousal, and disorganization thresholds	Direct feeling, wish, agency, contact, and movement in the task	Punitive superego, shame attack, and joy-to-collapse sequence
CBT/DBT/schema	Avoidance, safety behavior, detached coping, overcontrol, and mode shift	Hyperarousal, window of tolerance, emotion dysregulation, and physiological activation	Approach behavior, skill use, behavioral activation, and value-consistent action	Punitive parent mode, self-criticism, shame spiral, and defeat-based self-evaluation
Systemic/family–couple	Rigid role defense, triangulation, distancing move, and escalation-avoidance pattern	Interactional arousal, conflict load, and narrowing under relational stress	New transactional option, differentiated statement, repair move, and collaborative step	Internalized blame position, shame-based role capture, and self-invalidating stance
Contextual–relational	Experiential avoidance, fusion-protective move, and contact-avoidant pattern	Loss of workable contact, and over- or under-arousal relative to the moment	Openness, values-based step, present-moment contact, and fuller participation	Self-as-problem stance, shame contraction, and attacks on aliveness or need

PAD-S, Perceive–Assess–Dose–Safeguard; DEF, defensive/avoidant organization; ANX, anxiety/arousal and tolerance; PRO, progression toward direct experience and action; SUP, self-attack/shame processes.

### 5.2 Dyadic safety, rupture repair, and social embedding

Human functioning is interpersonal and embedded. A strictly individual state space is therefore insufficient for many clinically important phenomena. Let there be N interacting agents, such as patients, therapists, family members, or institutional actors. Each agent i has a latent state x_t_(;^i^) and a PAD-S projection z_t_(;^i^). A generic coupled system can be written as follows:

z_t+1_^(i)^ = F_i_(z_t_^(i)^, a_t_^(i)^, c_t_) + Σ_j_ ≠_i_ w_ij_ H_ij_(z_t_^(i)^, z_t_^(j)^, a_t_^(j)^, c_t_) + ξ_t_^(i)^,

where w_ij_ is a coupling weight and H_ij_ captures interaction mechanisms. This form is generic, but it makes explicit that the patient’s next state depends not only on their own current state and the action directed toward them but also on the states and actions of others. This is the minimum formal concession that a human-centered psychiatry should make to relational reality.

Clinically, one particularly important relational variable is felt safety. I therefore introduce an additional dyadic latent variable s_t_ ∈ [0,1] representing the degree of co-created safety or attachment security in the therapeutic dyad. Frederickson’s work is especially useful here because it frames alliance not as a static background condition but as an active, moment-to-moment accomplishment built through regulation, repair, and protection of emerging progress (12, 13). A simple update family is as follows:

s_t+1_ = s_t_ + α·repair_t_ − β·rupture_t_ + ω_t_.

**Figure 2:**
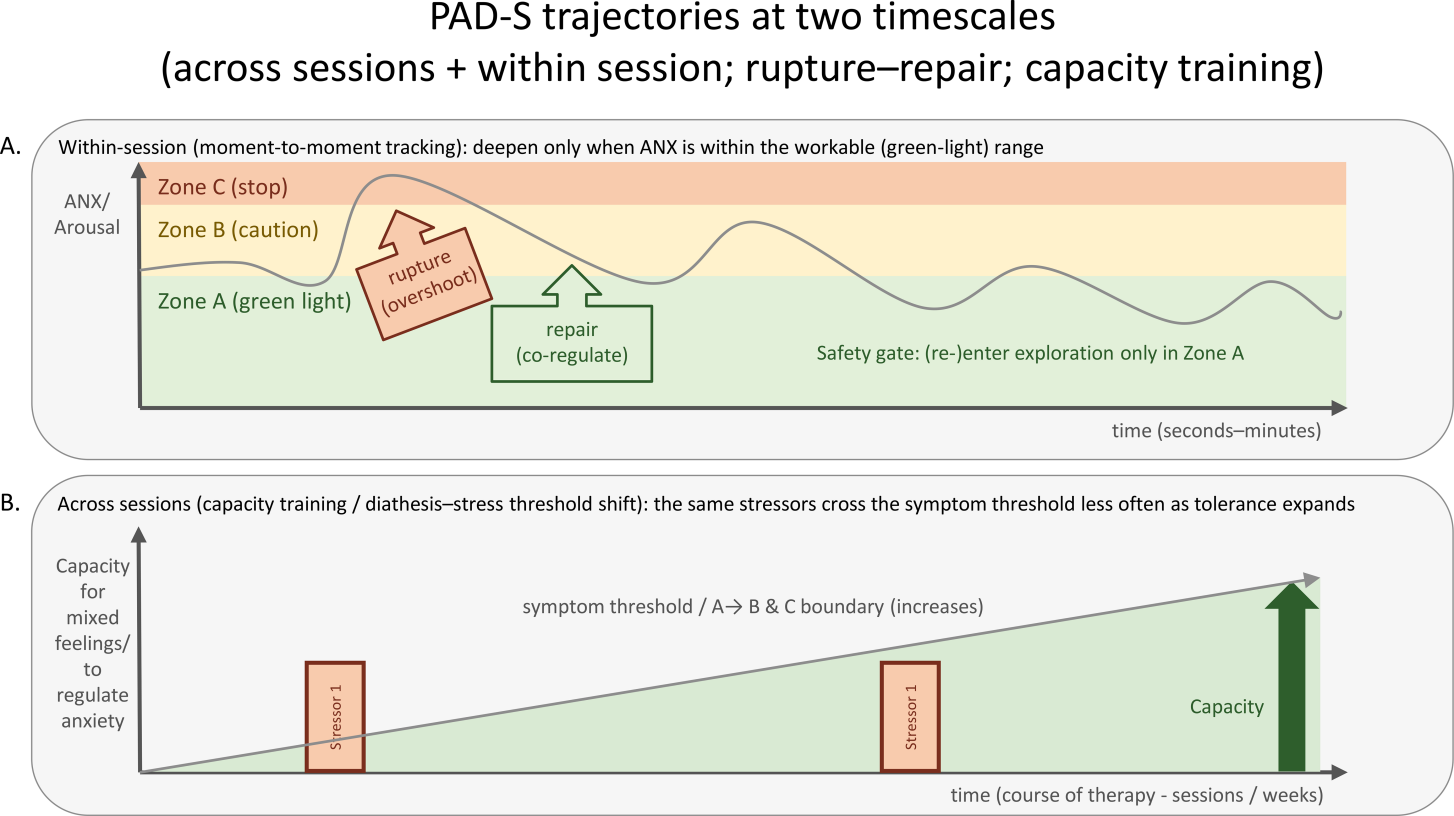
PAD-S trajectories at two timescales. **(A)** visualizes within-session safety gating: exploratory or deepening work is pursued only inside the individually workable range, and ruptures require repair before renewed challenge. **(B)** visualizes a longer-timescale capacity-training intuition: as tolerance expands, the same stressors cross the threshold into non-workable zones less often. PAD-S, Perceive–Assess–Dose–Safeguard.

**Figure 3:**
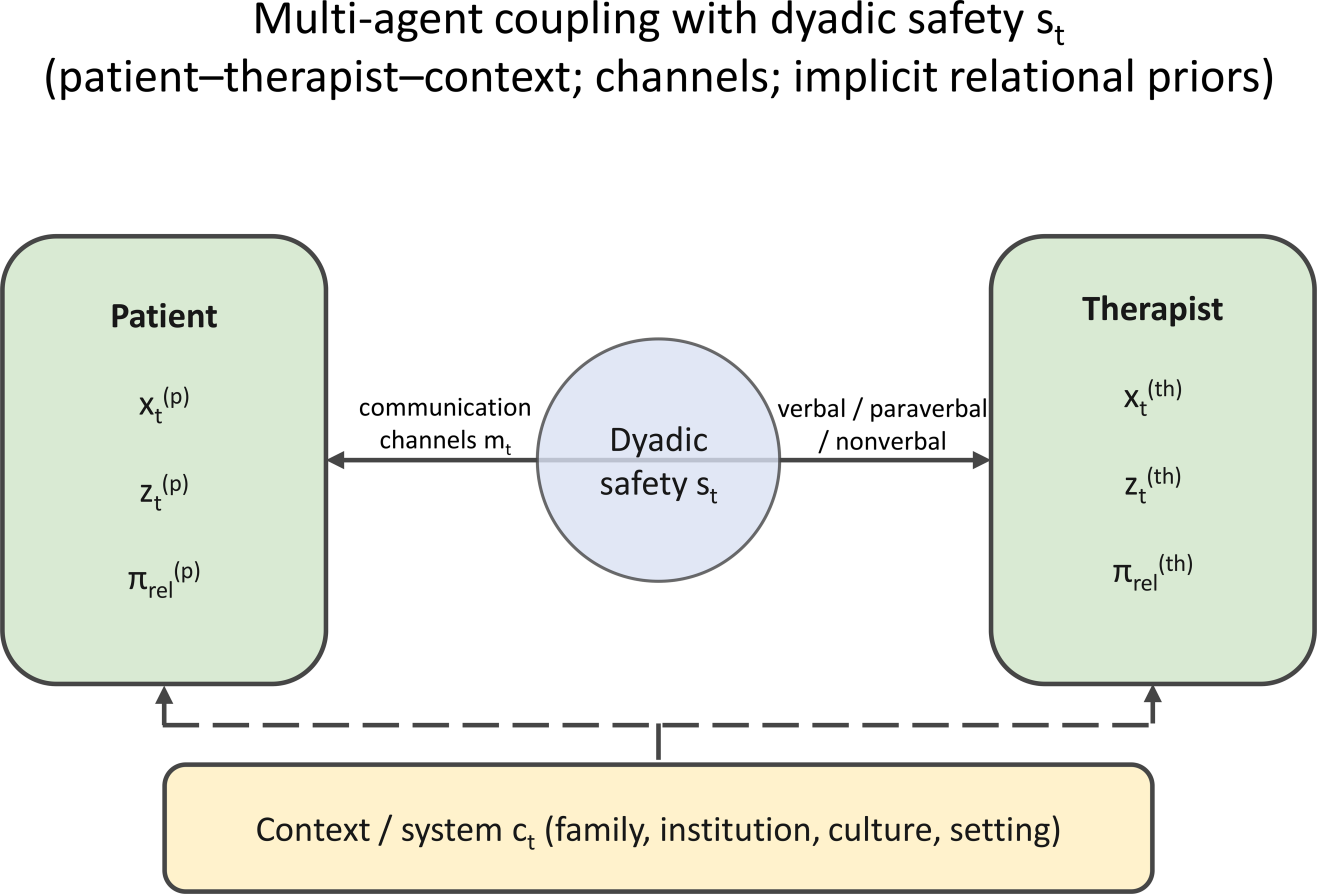
Multi-agent coupling with dyadic safety. The figure summarizes the patient–therapist–context extension, in which verbal, paraverbal, and non-verbal channels are embedded in a wider system and modulated by a dyadic safety variable.

Higher s_t_ expands what can be safely dosed; lower s_t_ narrows the admissible action set and increases the relevance of safeguard moves. In this way, dyadic safety links the representation layer to the clinically familiar logic of rupture, repair, co-regulation, and graded intensity without forcing that logic into a single therapeutic idiom.

(**Figures 2**, **3**) preserve two intuitions that are central to the present proposal. First, safety gating is a within-session phenomenon: the same patient can move from workable to non-workable states within seconds or minutes. Second, the representation layer is not limited to a dyad: it can be embedded in wider social, family, and institutional systems.

### 5.3 Function-first anchoring

A representation layer is not clinically credible if it cannot be connected back to function. For this reason, PAD-S/CSA is anchored here not only to process but also to functioning in the ICF/Mini-ICF-APP sense (16–18). The key claim is modest: changes in DEF, ANX, PRO, and SUP should become meaningful partly because they co-vary with changes in endurance, planning, decision-making, role performance, dyadic relatedness, assertiveness, or group participation.

This function-first stance has two advantages. It prevents the framework from becoming an inward-looking psychotherapy ontology, and it aligns the manuscript with rehabilitation- and participation-oriented psychiatric practice, where functioning under real-world constraints remains central (29). It also clarifies why the present article is interested in thresholds and dyadic safety: these are not merely elegant process concepts but candidates for explaining why the same diagnosis can support very different levels of practical functioning.

## 6 Staged validation roadmap

The framework should be judged less by rhetorical elegance than by whether it can survive a staged validation pipeline. The order matters. Computational use is not credible unless the human coding layer is itself stable, equation discovery is not interesting if the target representation is unreliable, and clinical usefulness is incomplete if the coordinates cannot be tied to functioning. The proposed pipeline, therefore, moves from coding reliability to construct/function linkage to transcript-level predictive operationalization under strict held-out designs and only then to sparse dynamic recovery.

### 6.1 Reliability as a prerequisite for computational use

A necessary condition for computational use is that independent raters can apply the episode-level coding with adequate agreement. For transcript or video material, the practical unit need not be a single speaking turn; often, the better unit is a brief episode line in which observation, threshold, and action are bundled into a clinically interpretable segment. The present proposal, therefore, recommends a reliability pilot in which a shared manual, a gold-standard training set, and structured adjudication precede formal double coding. This is consistent with the training logic of deliberate practice, which is increasingly recognized as important for psychotherapy skill development and supervision (27, 28).

For categorical node labels and threshold zones, weighted kappa-family statistics or related agreement coefficients with confidence intervals should be reported; if continuous anchors are introduced, intraclass correlations can be added. The key point is not to enforce a single statistic but to make the reliability problem visible and auditable. A representation layer that cannot be applied consistently by trained humans should not be promoted as a clinical AI interface.

### 6.2 Construct and functional linkage

Reliability alone is insufficient. The next question is whether the coordinates behave like the constructs they claim to represent. This can be approached through convergent and discriminant tests—for example, whether high-coded SUP co-occurs with clinically judged self-attack or shame collapse, whether high ANX predicts narrower workable ranges, or whether higher PRO is associated with more observable approach behavior and better session-level task engagement.

Functional linkage is equally important. Mini-ICF-APP and related ICF-based formulations provide a realistic bridge here because they are already used to describe role capacity, endurance, planning, decision-making, dyadic relatedness, and participation (16–18). If PAD-S trajectories never connect to functioning, then the representation may still be academically interesting but clinically underpowered. Conversely, stable links to function would strengthen the claim that the coordinates are not merely theoretical conveniences. **Table 4** summarizes operational anchors linking the four PAD-S coordinates to observable cues and likely functional relevance.

**Table 4:** Operational anchors linking the four PAD-S coordinates to observable cues and likely functional relevance.

Coordinate	Clinically salient question	Observable cues (illustrative, not exhaustive)	Likely functional linkage
DEF	How is access to conflict, need, or reality being avoided or reorganized?	Topic shifts, generalities, over-explaining, joking, rigid role moves, mode switches, and distancing maneuvers	Planning failures, stalled decisions, reduced task engagement, and repetitive relational loops
ANX	How much arousal is present, and how workable is it now?	Breath-holding, muscular tension, gastrointestinal (GI) activation, narrowing, cognitive-perceptual disruption, and overload/underload	Reduced endurance, impaired concentration, and instability under interpersonal or role stress
PRO	Is there usable forward movement toward contact, agency, or action?	Need articulation, clearer focus, approach behavior, workable contact, valued step, and repair initiative	Improved assertiveness, role participation, collaboration, and behavioral follow-through
SUP	Is progress being followed by shame, self-attack, or punitive collapse?	Head drop, contemptuous self-talk, moralized self-judgment, joy-to-collapse sequence, and attacks on need or success	Withdrawal, avoidance of visibility, loss of gains, and collapse of self-care or interpersonal participation

PAD-S, Perceive–Assess–Dose–Safeguard; DEF, defensive/avoidant organization; ANX, anxiety/arousal and tolerance; PRO, progression toward direct experience and action; SUP, self-attack/shame processes.

### 6.3 Predictive operationalization before equation discovery

Before sparse discovery, the representation layer should first survive a simpler predictive test. The question at this stage is not whether PAD-S/CSA already yields elegant equations, but whether coded trajectories contain enough stable signal to support transcript- or case-held-out prediction of a next-state or next-action structure. This stage functions as an observability and learnability check for the representation.

Implementation is intentionally left open at this stage. A later empirical paper should compare simple non-sequential baselines with sequential or state-space approximators using transcript- or case-held-out designs rather than shuffled turns. Load-bearing outputs would include transparent per-label and macro-averaged performance, confusion patterns, and sensitivity analyses for ambiguous or mixed-coded episodes.

The aim is not to claim a final predictive architecture here but to establish whether PAD-S/CSA can be operationalized without leakage or convenience splitting. Only if such predictive operationalization proves stable does sparse equation discovery become informative. Otherwise, one risks searching for elegant equations in a representation that has not yet demonstrated transcript-level predictive adequacy.

### 6.4 Sparse equation discovery (including SPICE-type workflows)

Once reliable coded trajectories and predictive operationalization exist, the next step is to test whether the representation admits a sparse, interpretable transition structure. One candidate family of methods for this stage is SPICE, which combines latent-dynamics modeling with sparse equation extraction (24). Related Sparse Identification of Nonlinear Dynamics (SINDy)-style workflows could play a similar role (25). The point of naming SPICE here is not to imply current implementation but to identify the kind of later method that would make the architecture mechanistically testable.

In PAD-S/CSA terms, such methods would take time-indexed sequences of z_t_, τ_t_, a_t_, and c_t_—and optionally s_t_ and channel metadata—and search a clinically interpretable candidate library for a sparse transition structure. Evaluation should hold out entire transcripts or cases and report both predictive generalization and term stability across folds or bootstraps.

Sparse equation discovery is relevant here only as a later test of representational adequacy. If no stable and clinically interpretable sparse structure survives transcript-level hold-out, or if equally accurate but structurally incompatible solutions proliferate, the representation claim is weakened. If a stable, sparse structure does emerge, later work would have moved beyond generic prediction toward candidate mechanisms.

### 6.5 Current empirical status and evidential boundaries

Prior PAD-S and CSA publications established the basic clinical vocabulary, the episode-line format, the transcript-oriented documentation logic, and the function-linked coding agenda (8, 9). They did not, however, make representational adequacy itself the primary analytic target. The present theory article adds no new dataset, no new reliability estimate, no new transcript-classification result, and no discovered equation.

This evidential separation is deliberate. A theory paper should not borrow authority from analyses that are not fully reportable here. Its contribution is to isolate the architectural claim, specify a restricted model family, and define the order in which later empirical work would need to test that claim.

A later empirical sequel should report the segmentation unit, coder training and adjudication, agreement statistics, transcript- or case-held-out design, candidate library, and governance procedures in full. Until such results exist, PAD-S/CSA should be read as a falsifiable proposal for a shared representation layer rather than as demonstrated computational performance.

### 6.6 Governance, privacy, and human-final use

Because the intended use case is high-stakes mental health care, governance must be built into the framework rather than appended as an afterthought. The PAD-S/CSA representation layer should be treated as decision support, not autonomous decision-making. Human final judgment remains essential at every step: in coding, in model review, and in any clinical use of outputs. The coordinates should not be treated as hidden truths about the person, should not override patient self-description, and should not be used to prescribe interventions automatically. This stance is consistent with human-centered AI guidance from the World Health Organization and with the risk-based obligations articulated in the EU AI Act (4, 5, 30).

For transcript-based pilot work, the governance baseline is comparatively simple but non-negotiable: use of pre-existing anonymized transcripts, processing on local institutional servers, restricted access, no cloud dependency for the pilot stage, and explicit separation between research coding and clinical decision authority. A representation layer that is meant to preserve human dignity cannot be built on opaque data handling. **Table 5** summarizes the staged validation and governance roadmap.

**Table 5:** Minimal staged validation roadmap for PAD-S/CSA as a shared representation layer.

Layer	Question	Typical data	Primary metric(s)	What would count against the framework?
Reliability	Can trained raters code node, threshold, and action/dose consistently?	Transcripts, audio, or video with shared manual	Weighted kappa-family coefficients; intraclass correlation coefficients (ICC) where applicable; confidence intervals	Agreement remains poor despite training and adjudication support
Construct linkage	Do the coordinates behave like the processes they claim to represent?	Coded episodes plus clinician ratings/independent process measures	Convergent and discriminant associations	No coherent relation between coded PAD-S states and independently judged process phenomena
Functional linkage	Do coded trajectories connect to functioning?	PAD-S episodes plus Mini-ICF-APP/ICF-oriented ratings	Associations with endurance, planning, role participation, and dyadic relatedness	No meaningful connection to functioning or participation
Predictive operationalization	Can held-out models learn stable transition structure from coded PAD-S trajectories?	Time-indexed PAD-S episodes; explicit transcript/case hold-out; documented segmentation unit	Per-label precision/recall/F1; macro-F1; confusion matrices; baseline comparison; sensitivity analyses	Performance collapses under transcript-level hold-out, depends on leakage, or stays near weak baselines
Sparse dynamics	Can sparse equations predict state transitions out of sample?	Time-indexed z_t_, τ_t_, a_t_, and c_t_ (and optionally s_t_); documented segmentation unit	Transcript-/case-held-out prediction; term stability across folds/bootstraps; model sparsity; interpretability	Only transcript-specific overfit, unstable equations, or mutually incompatible sparse solutions emerge
Governance	Can the system remain human-centered and rights-compatible?	Protocol, data flow, server access, and audit trail	Human-final use; local processing where required; restricted access; documentation; audit trail	Opaque processing, no auditability, or drift toward automation-first deployment

PAD-S, Perceive–Assess–Dose–Safeguard; CSA, Conflict-Square Algorithm.

## 7 Discussion

This article makes a narrower and, for that reason, stronger claim than a unified psychotherapy theory or a finished computational psychiatry model. PAD-S/CSA is presented here as a candidate shared representation layer rather than as an all-purpose ontology or a mandatory intervention algorithm. That repositioning matters because it aligns the ambition of the paper with what the formalism and the evidence can currently support.

This reframing also clarifies the place of psychotherapy pluralism. A shared representation layer is valuable precisely because different schools do not collapse into one another. If all therapeutic orientations already described the same phenomena in the same terms, there would be no representational problem to solve. At the same time, if they shared nothing, translation would be impossible. The workable middle position is that overlap and difference coexist. The four-coordinate PAD-S proposal is an attempt to mark some of that overlap without denying theoretical and technical specificity.

Several limitations remain. First, the four coordinates are justified here by design rationale and clinical action relevance rather than by definitive psychometric closure. Future work may show that one of the coordinates should be split, merged, or supplemented. Second, the present article contributes architecture rather than an empirical test: it reports no new reliability estimate, transcript-classification result, or discovered equation. Third, some clinically crucial phenomena—identity diffusion, reflective function, attachment narrative organization, culture-specific meaning, institutional violence, and longer-horizon life structure—may require additional slow-timescale variables beyond the present low-dimensional layer.

Even with those limits, the framework is useful if it improves clarity at the interface between psychotherapy, functioning, and computational modeling. It offers one way of connecting momentary process to role-relevant functioning, comparing schools without forcing theoretical unification, and moving from heterogeneous observation streams toward interpretable mechanistic hypotheses rather than only black-box prediction.

The decisive next step is therefore empirical, and the division of labor should remain explicit. The present paper defines the candidate shared representation layer, its non-goals, and the order of falsification. A later empirical paper should test whether episode coding is reliable, whether coded trajectories support transcript-level predictive operationalization under strict held-out designs, and only then whether sparse equations recovered from real transcripts remain stable out of sample. If these steps succeed, the case for PAD-S/CSA as a shared representation layer strengthens. If they fail, the framework should be revised or abandoned.

The appeal of sparse discovery in this setting is therefore not anti-machine-learning. It is pro-interpretability. In a high-stakes domain, accurate black-box prediction is often less useful than a somewhat simpler model whose transition terms can be inspected, disputed, and revised by clinicians and patients (26).

A related conceptual point concerns the gap between information and human meaning. A signal can be highly informative in the Shannon sense and still be clinically shallow if it fails to preserve values, agency, or real-world role consequences (31). A human-centered representation layer should therefore be judged not only by compressibility or prediction but by whether the compressed variables remain meaningful in lived clinical practice.

## Data Availability

The original contributions presented in the study are included in the article/supplementary material, further inquiries can be directed to the corresponding author/s.
